# The role of orgasm in the development and shaping of partner preferences

**DOI:** 10.3402/snp.v6.31815

**Published:** 2016-10-25

**Authors:** Genaro A. Coria-Avila, Deissy Herrera-Covarrubias, Nafissa Ismail, James G. Pfaus

**Affiliations:** 1 Centro de Investigaciones Cerebrales, Universidad Veracruzana, Xalapa, Mexico; 2 School of Psychology, University of Ottawa, Ottawa, Canada; 3 Center for Studies in Behavioral Neurobiology, Concordia University, Montréal, Canada

**Keywords:** orgasm, ejaculation, partner preference, pair bonding, opioids, dopamine, sexual reward

## Abstract

**Background:**

The effect of orgasm on the development and shaping of partner preferences may involve a catalysis of the neurochemical mechanisms of bonding. Therefore, understanding such process is relevant for neuroscience and psychology.

**Methods:**

A systematic review was carried out using the terms Orgasm, Sexual Reward, Partner Preference, Pair Bonding, Brain, Learning, Sex, Copulation.

**Results:**

In humans, concentrations of arousing neurotransmitters and potential bonding neurotransmitters increase during orgasm in the cerebrospinal fluid and the bloodstream. Similarly, studies in animals indicate that those neurotransmitters (noradrenaline, oxytocin, prolactin) and others (e.g. dopamine, opioids, serotonin) modulate the appetitive and consummatory phases of sexual behavior and reward. This suggests a link between the experience of orgasm/sexual reward and the neurochemical mechanisms of pair bonding. Orgasm/reward functions as an unconditioned stimulus (UCS). Some areas in the nervous system function as UCS-detection centers, which become activated during orgasm. Partner-related cues function as conditioned stimuli (CS) and are processed in CS-detector centers.

**Conclusions:**

Throughout the article, we discuss how UCS- and CS-detection centers must interact to facilitate memory consolidation and produce recognition and motivation during future social encounters.

It isn't surprising that he should prefer his mistress, whose features, to him, offer a hundred units. Even little facial imperfections on other women, such as smallpox scar, touch the heart of a man in love, inspiring a deep reverie; imagine the effect when they are on his mistress's face. The fact is, that pockmark means a thousand things to him, mostly lovely and all fully interesting. The sight of a scar, even on another woman's face will strongly remind him of all these things.

(Stendhal, 1822/2013). *De l'amour* (‘On love’), Chapter 17 (p. 53)

## Introduction

The effect of orgasm on the development and shaping of partner preferences involves catalysis of the neurochemical mechanisms of bonding (Coria-Avila et al., 2014). In men and women, concentrations of arousing neurotransmitters, like noradrenaline, and potential bonding neurotransmitters, like oxytocin (OT) and prolactin (PRL), increase during orgasm in the cerebrospinal fluid and the bloodstream (Carmichael et al., 1987; Exton et al., 1999; Kruger, Haake, Hartmann, Schedlowski, & Exton, 2002; Kruger et al., 2003; Murphy, Checkley, Seckl, & Lightman, 1990). Similarly, studies in animals indicate that neurotransmitters such as noradrenaline, oxytocin, prolactin and others (dopamine, opioids, serotonin, etc.) modulate the appetitive and consummatory phases of sexual behavior and the reward states that they induce (Pfaus, Damsma, et al., 1990; Pfaus & Phillips, 1991; Snowdon & Ziegler, 2015; Szechtman, Hershkowitz, & Simantov, 1981; Todd & Lightman, 1986; van Furth, Wolterink, & van Ree, 1995). This suggests a link between the experience of orgasm/sexual reward and the neurochemical mechanisms of pair bonding, something alluded to in Stendhal's Principle concerning how love develops in couples that experience physical closeness after orgasm (Stendhal, 1822/2013), and later in John Money's concept of how ‘love maps’ develop (Money, 1997).

An orgasm is a powerful stimulus that through positive reinforcement may increase the chances that copulation will occur again with that partner. Subsequently, partner-related cues experienced in the presence of sexual reward come to elicit a representation of that reward, and thereby become desired features that identify the partner as the beloved, or are chosen to the exclusion of other features possessed by other potential mates. This process is especially potent during an individual's first sexual experience (Pfaus, Kippin, & Centeno, 2001) but may be additive throughout the lifespan, such that several love maps develop, all of which are made up of attractive and preferred partner-related cues (Money, 1997). This phenomenon appears to be highly sensitive to the situations in which they are first experienced (Quintana-Zunino, Primeau, & Pfaus, 2015), and perhaps is experienced differently by individuals according to the degree of reward, attention, and bonding capacity they are born with. For example, in polygamous species like rats, sexual reward may induce brain activation that results in short-lasting preferences, whereas in other species like monogamous prairie voles, sexual reward can result in long-lasting preferences. The aim of this review is to provide information on the behavioral evidence and the neural correlates underlying orgasm-induced partner preferences. We begin by describing orgasm and sexual reward, and changes in partner preference after sex. This is followed by a discussion of the neuroendocrine changes during orgasm in humans or during sexual reward in animal models and how those changes can explain the development and shaping of partner preferences.

## Orgasm and sexual reward

In humans, an orgasm has been defined as ‘a variable, transient peak sensation of intense pleasure, creating an altered state of consciousness, usually accompanied by involuntary, rhythmic contractions of the pelvic striated musculature, often with concomitant anal contractions and myotonia that resolves the sexually-induced vasocongestion, usually with an induction of well-being and contentment’ (Meston, Levin, Sipski, Hull, & Heiman, 2004). The eruption of euphoric pleasure at orgasm is often followed by sedation, satiety, and feelings of relief and enjoyment. Because these feelings can only be determined by verbal expression in humans, it is difficult to assess them in other species unless a parallel can be drawn either by analogy or homology. Such an analogy exists if we consider orgasm to be one of several states of sexual reward. This is important because studies in laboratory animals using the conditioned place preference paradigm (CPP) confirm that males experience sexual reward following ejaculation (post-orgasmic state) and females during paced copulation at preferred rates and timed clitoral stimulation (pre- and post-orgasmic) (Agmo & Berenfeld, 1990; Parada, Chamas, Censi, Coria-Avila, & Pfaus, 2010; Paredes & Alonso, 1997; Paredes & Martinez, 2001). Thus, although orgasm *per se* has never been reported in animals as we know it in humans, scientists can infer its existence based on the pre- and post-orgasmic states reported in humans and the reward-based learning observed in animals. Indeed, the study of such homologies and analogies can provide information that we cannot ascertain from clinical work only.

Sexual reward itself can be defined as a state of pleasure in which sexual behaviors that precede it are positively reinforced as operants, and sexual stimuli (both contextual and partner-related) are associated in a predictive Pavlovian manner (Crawford, Holloway, & Domjan, 1993; Pfaus et al., 2012). In this way, an animal's sexual arousal, desire, and partner preferences are primed by the presence of cues associated previously with sexual reward, and sexual behaviors that generate the reward state become sensitized across an acquisition period. Ejaculation in men, for example, is typically associated with orgasm (Kinsey, Pomeroy, & Martin, 1948), although ejaculation can occur with a diminished or absent orgasm, for example, in some instances of premature or rapid ejaculation (Sotomayor, 2005), and orgasm can occur without ejaculation (Roehrborn, Kaplan, Lepor, & Volinn, 2011). In most male rats, ejaculations, but not penile intromissions, are necessary for the development of a CPP. This indicates that in male rats, ejaculation produces a state of reward that is naturally associated with contextual or environmental cues (Agmo & Berenfeld, 1990). However, in male rats that have never ejaculated, intromissions alone induce a weaker CPP, suggesting that intromissions without ejaculation can be experienced as rewarding based on a hierarchy that depends upon prior sexual experience (Tenk, Wilson, Zhang, Pitchers, & Coolen, 2009). In a Pavlovian account, the distinctive side of the CPP box associated with ejaculation functions as a contextual conditioned stimulus (CS) that earns incentive value through its association with the state induced by ejaculation that functions as the unconditioned stimulus (UCS). Ejaculation-induced CPP in rats can be prevented by injections of the opioid receptor antagonist naloxone (Agmo & Berenfeld, 1990) and by dopamine type 1 receptor (D1) antagonists (Dominguez-Salazar, Naser, & Velazquez-Moctezuma, 2014). Thus, D1 receptor activation prior to ejaculation and opioid receptor activation after ejaculation are both critical for sexual reward in male rats.

Female hamsters and quails develop a CPP for distinctive sides of a chamber, where they experienced sex with a male (Gutierrez & Domjan, 2011; Meisel & Joppa, 1994). Other studies in female rats have shown that they can develop a CPP if are placed within the distinctive side immediately after sex, namely after paced copulation (Paredes & Alonso, 1997; Paredes & Martinez, 2001). Accordingly, sex or the consequences of sex can induce a CPP. When females pace copulation, they can regulate the timing of successive intromissions (Erskine, 1989). Control over the rate of clitoral stimulation (CLS) (Parada et al., 2010) and vaginocervical stimulation (VCS), received from mounts and intromissions, activate the appropriate appetitive and neuroendocrine systems to facilitate sexual reward and reproduction (Adler, 1969; Diamond, 1970; Georgescu, Sabongui, Del Corpo, Marsan, & Pfaus, 2009; Pfaus, Manitt, & Coopersmith, 2006; Pfaus, Marcangione, Smith, Manitt, & Abillamaa, 1996; Terkel & Sawyer, 1978). In rats copulating in large open spaces, pacing occurs when the female solicits the male and runs away at her preferred intervals, receiving genitosensory stimulation when she stops and assumes a lordosis posture (McClintock, 1984). Pacing chambers have been developed for laboratory use. For example, bi-level pacing chambers have two levels and sets of ladders on the sides leading up to ramps between each. Females can regulate their copulatory intervals with males by running from level to level, forcing males to chase them (Mendelson & Pfaus, 1989; Pfaus, Mendelson, & Phillips, 1990; Pfaus, Smith, Byrne, & Stephens, 2000; Pfaus, Smith, & Coopersmith, 1999). Unilevel pacing chambers have a Plexiglas partition in the middle with one or more small holes cut out of the bottom that are large enough to allow the female to pass from one side without a male to the other side with a male, but too small to allow the male to pass from side to side (Paredes & Alonso, 1997; Paredes & Martinez, 2001). Thus, the female regulates her sexual contact with the male by running to and from his side of the chamber. Likewise, unilevel ‘racetrack’ chambers have a Plexiglas divider that extends through the middle of the chamber lengthwise, with an area cut out of each side. Females can pace by running from side to side, allowing males to chase them (Pfaus et al., 2009). Non-paced copulatory conditions can be contrasted in the same chambers without the partition. In all of the studies noted above, female rats find paced copulation more rewarding than non-paced copulation (as observed with CPP). Furthermore, systemic or intracerebral injections of naloxone, but not dopamine antagonists, prior to pacing trials prevent the development of CPP, indicating that sexual reward induced by pacing in female rats is mediated mainly by endogenous opioids (Garcia Horsman & Paredes, 2004; Garcia-Horsman, Agmo, & Paredes, 2008; Paredes & Martinez, 2001), although in other rodent species like syriam hamsters, treatment with D2 antagonists can readily disrupt sex-induced CPP (Meisel, Joppa, & Rowe, 1996). Interestingly, naloxone also reduces the subjective pleasure experienced at human orgasm (Murphy et al., 1990), suggesting that, like in animals, the rewarding experience is mediated by endogenous opioids. In addition, animals share some other orgasm-like responses with humans that may occur during the experience of sexual reward such as rhythmic contractions of the pelvic musculature (Carro-Juarez & Rodriguez-Manzo, 2000) and uterus (Toner & Adler, 1986), increased blood levels of PRL after mounts with intromission in females (Helena, McKee, Bertram, Walker, & Freeman, 2009; Yang, Lee, & Voogt, 2000) and after ejaculation in males (Hernandez et al., 2006), analgesia in both females and males (Gomora, Beyer, Gonzalez-Mariscal, & Komisaruk, 1994; Gonzalez-Mariscal, Gomora, Caba, & Beyer, 1992), and most importantly similar brain activation (Georgiadis, Kringelbach, & Pfaus, 2012).

## ‘Pre- and post-orgasmic learning’ of partner preference

CPP and conditioned approach or behavior are consistent with the idea that animals will approach and prefer stimuli that have been paired with reward (Nader, Bechara, & van der Kooy, 1997). This indicates a search strategy based on past experiences, so that animals have greater probability of coming into contact with stimuli that induce positive hedonic effect, and that, if found on partners, may represent increased fitness (Panksepp, Knutson, & Burgdorf, 2002). In male rats, for example, neutral odors associated with the post-ejaculatory reward state facilitate motivation for a scented female partner in future encounters (Kippin, Talinakis, Chattmann, Bartholomew, & Pfaus, 1998). In that study, one group of males (the paired group) was trained to associate an almond or lemon odor (painted on the back of a female's neck and anogenital region) with copulation to ejaculation in bi-level chambers over several trials. A second group (the unpaired group) was allowed to copulate to ejaculation with unscented females in the same type of chamber. On a final test, the males had access to two sexually receptive females in a large open field: one scented with the odor and the other unscented. Males in the paired group chose to ejaculate first and more times with the scented female, whereas males in the unpaired group ejaculated more times with the unscented female. In subsequent experiments, it was demonstrated that this ‘conditioned ejaculatory preference’ requires that the male not simply intromit or ejaculate, but that he must be with the female during his postejaculatory interval (PEI), the refractory period prior to the resumption of another copulatory bout (Kippin & Pfaus, 2001a). This phenomenon was referred to as ‘postejaculatory learning’ (Kippin & Pfaus, 2001b). Indeed, copulating with an unscented female, but having her replaced with a scented female during the PEI, was sufficient to induce a conditioned ejaculatory preference for the scented female. More recently, it was shown that systemic treatment with naloxone, but not the general D1/D2 antagonist flupenthixol, disrupted the development of conditioned ejaculatory preference (Ismail, Girard-Beriault, Nakanishi, & Pfaus, 2009). Accordingly, during the PEI, males undergo neuroendocrine changes (e.g. opioid release) that trigger a neurochemical cascade that helps consolidate learning about the female they are with (Pfaus et al., 2001). This occurs via Pavlovian conditioning during the PEI that links a neutral stimulus on the partner to the sexual reward induced by ejaculation (Kippin & Pfaus, 2001a, b; Kippin et al., 1998).

Although the PEI is the main component that facilitates the development or shaping of partner preference, evidence indicates that what happens before ejaculation (pre-orgasm) may be also important for the whole experience of sexual reward and perhaps for the shaping of partner preferences. For example, rats that ejaculate too fast (i.e. treated with the serotonin 1A receptor agonist 8-OH-DPAT) or with no preceding intromissions are said to have a facilitated (rapid) ejaculation but not a facilitated sexual motivation or desire. Those rats fail to show ejaculation-induced CPP (Camacho, Castro, Hernandez, & Paredes, 2007), suggesting that an ejaculation that comes too quickly without increasing levels of arousal during copulation is either not experienced as rewarding or may not be processed as a salient-enough UCS. Ismail and colleagues found that males trained in unilevel pacing chambers with scented females under the condition of a one-hole divider developed significant conditioned ejaculatory preferences for a scented female over an unscented female. However, males trained under the condition of a four-hole divider did not develop a significant conditioned ejaculatory preference, despite ejaculating on all training trials (Ismail, Gelez, Lachapelle, & Pfaus, 2009). Why this difference? Relative to males in the four-hole condition, males in the one-hole condition displayed significantly more intromissions prior to each ejaculation and had longer inter-intromission intervals owing to the fact that they had to wait longer for the female to return to their side. This suggests that those males, like the males that have to chase females in bilevel chambers, were more sexually aroused than males in the four-hole condition. This is reminiscent of men for whom high levels of arousal during infatuation facilitate memory and attention toward partner-related cues (Langeslag, Olivier, Kohlen, Nijs, & Van Strien, 2015). Thus, although the ejaculatory reward state is necessary, it is not sufficient – for a male to develop a strong partner preference, the PEI (post-orgasm) must be preceded by sufficient amounts of sexual desire and/or arousal (pre-orgasm).

Female rats also develop a partner preference for males that bear conditioned cues paired with timed CLS (Parada, Abdul-Ahad, Censi, Sparks, & Pfaus, 2011) or pacing-related sexual reward (Coria-Avila, Ouimet, Pacheco, Manzo, & Pfaus, 2005). For example, the study of Parada et al. (2011) showed that female rats can display a conditioned partner preference for males scented with an odor previously paired alone with timed CLS (not on a male). However, when timed CLS occurs in contingency with the presence of a scented but inaccessible male, females do not learn to prefer him. The authors argued that the latter occurs as a result of a sexual inhibitory state. Furthermore, Coria-Avila et al. (2005) showed that pigmented Long-Evans female rats prefer unfamiliar Long-Evans males scented with an almond odor previously associated with paced copulation but not when the odor is associated with non-paced copulation or is randomly associated with both paced and non-paced copulation (Coria-Avila et al., 2005). In subsequent experiments, it was shown that Albino Wistar female rats also display a partner preference for unfamiliar Wistar or Long-Evans males if the strain was previously associated with sexual reward induced by pacing (Coria-Avila et al., 2006). In general, females expressed their partner preference with more proceptive precopulatory behaviors, such as solicitations and hops and darts, and also chose the pacing-related male to receive their first ejaculation. Injections of naloxone prior to each paced copulation trial prevented the development of partner preferences, such that females failed to prefer the male that bore the conditioned odor or strain cue paired with sexual reward (Coria-Avila, Solomon, et al., 2008). Furthermore, systemic injections of the dopamine receptor antagonist flupenthixol prior to paced copulation prevented only the development of conditioned partner preference for the male bearing the odor but not the conditioned preference for strain (Coria-Avila, Gavrila, et al., 2008). Thus, based on the results of male and female rats, it is suggested that endogenous opioids are necessary for the experience of sexual reward to be paired with any kind of features detected on a partner. However, dopamine may be required only to learn the association of sexual reward with a neutral olfactory cue (like almond odor) but may not be mandatory for the association of reward with more ‘natural’ strain-related cues in polygamous or promiscuous species.

Copulation also facilitates pair bonding in monogamous prairie voles (Williams, Catania, & Carter, 1992). Pair bonds are observed when an individual has the choice of two partners, one familiar, with whom copulation occurred previously, and one novel. A bonded vole will choose the familiar one to spend more time, copulate, and reproduce sometimes for life (Getz, McGuire, Pizzuto, Hofmann, & Frase, 1993). As in rats, systemic treatment with the opioid receptor antagonist naltrexone prevents the development of pairbonds (Burkett, Spiegel, Inoue, Murphy, & Young, 2011). Interestingly, dopamine plays an important role in the development of preferences in this monogamous species (Young & Wang, 2004). We have previously discussed the possibility that a bonded vole remains monogamous for life because of the constant positive reinforcement from the partner during social contact and sexual reward (i.e. opioid-induced). However, it is also possible that the monogamous preference is maintained via increased desire, attention, or expectation of reward that depends on dopamine activity. Accordingly, the specific features of the partner (e.g. olfactory signature) may become conditionally preferred and reinforced by concomitant social stimulation and sexual reward (Coria-Avila et al., 2014).

One of the hallmarks of a monogamous sexual strategy is mate guarding in the presence of a competitor. Despite female rats being referred to as ‘promiscuous’ (Barnett, 1963; McClintock, 1984; Parker, 1984), they display a pattern of mate-guarding behavior if their first experience of sexual stimulation occurs under optimal paced conditions with the same male, and they are subsequently placed into an open field with that male and a sexually receptive competitor female (Holley, Shalev, Bellevue, & Pfaus, 2014). This pattern is characterized by three behaviors: hovering and Presenting (HP), where the female stays near or next to the male, often assuming a pre-lordosis presenting posture; conspecific blocking (CB), where the female places her body between the male and the competitor female should the competitor approach the two; and female–female mounting (FFM), where the female mounts the competitor female several times in an agonistic dominance display if the competitor solicits the male. Over the course of several open field trials, FFM sensitizes such that the female will engage in it immediately upon presentation of the conspecific female. Over time, this results in the competitor staying in a corner for the duration of the test, allowing the female and male to copulate freely without interference. Importantly, females given their first sexual experiences with different males each time do not show these patterns of behavior, nor do females paired with one particular male but given access to a different male in the open field tests. Treatment with OT during the female's first paced sexual experience with the male potentiates HP selectively, whereas treatment with arginine vasopressin (AVP) potentiates CB selectively (Holley et al., 2015). Preliminary evidence suggests that FMM is potentiated by the dopamine agonist apomorphine. Thus, the three neurotransmitter systems that are involved in monogamous sexual behavior in prairie voles are involved in the display of conditioned mate-guarding behavior in female rats. These behaviors are not displayed if the female is treated with naloxone, or with a lysine-specific demethylase inhibitor, suggesting that opioid reward is critical and that the behavior results from epigenetic changes (Holley, 2015).

## Neural pathways that mediate orgasm-induced partner preferences

**Fig. 1:**
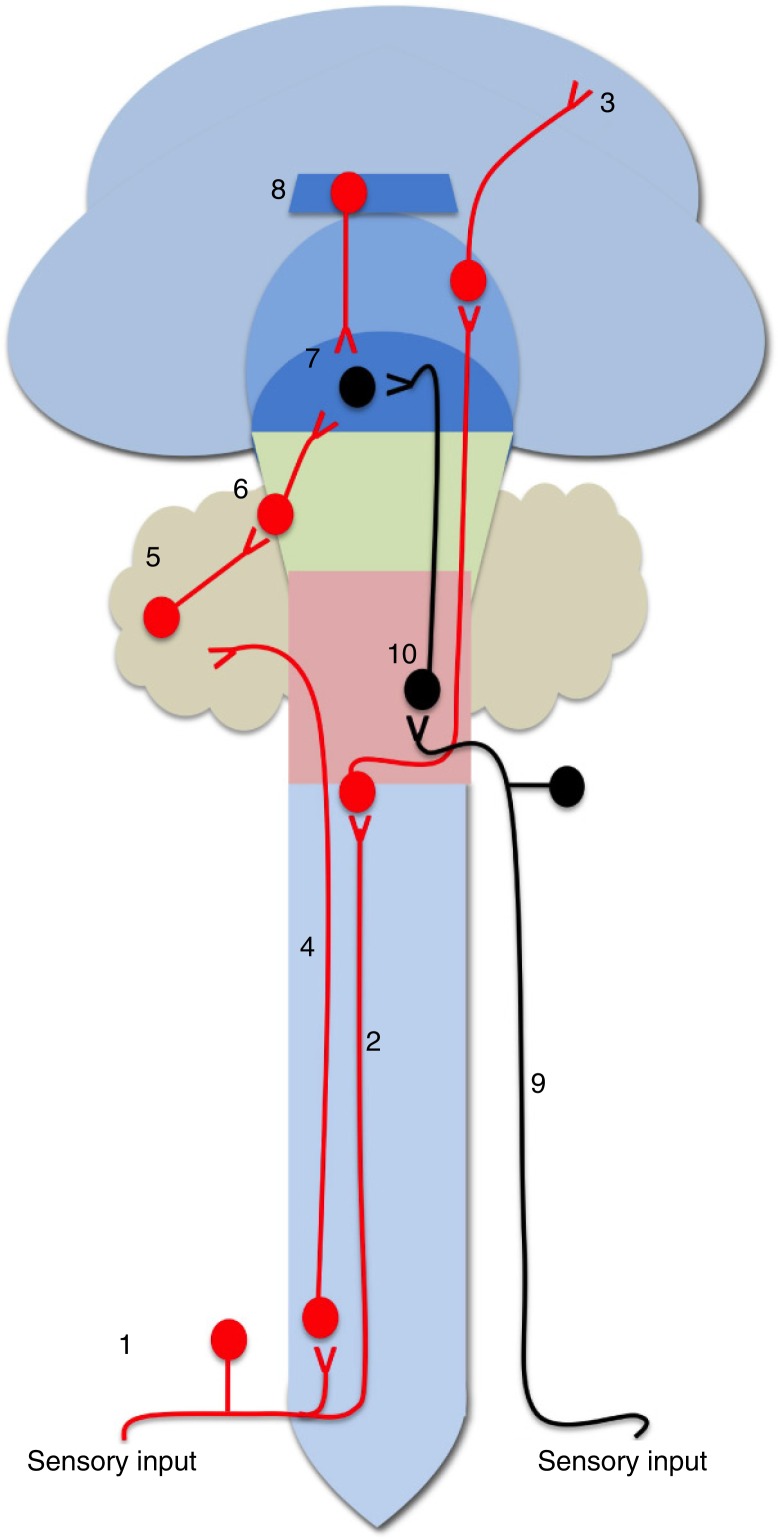
Peripheral and central neurocircuitry involved in the development and shaping of orgasm-induced partner preferences. For full explanation, see Section ‘A model’. In RED, we depict the classic sensory input pathway: (1) peripheral nerves, (2) hindbrain, thalamus, and (3) sensory cortex (where orgasm is experienced). At the same time, (4) spino-cerebellar pathway projects information to (5) cerebellum, whose main output is via its deep nuclei, and also projects to (7) hypothalamus. (8) During orgasm, the hippocampus may process explicit and implicit memories. In BLACK, we depict a secondary ‘sensory’ pathway that involves (9) the vagus nerves and (10) hindbrain (nucleus of the tractus solitarious, periaqueductal gray matter), and from there to (7) hypothalamus.

In order to facilitate the development and shaping of partner preferences, an orgasm-like state must be interpreted as a rewarding UCS in brain areas related to the processing of sensory information but also must activate areas that mediate social recognition, learning, and desire in future encounters (Coria-Avila et al., 2014). In male and female rodents, one main pathway from the genitals may include the pudendal, pelvic, and hypogastric nerves (Berkley, Robbins, & Sato, 1988; Giuliano & Rampin, 2004; Ueyama, Arakawa, & Mizuno, 1987; Yucel & Baskin, 2004). In the female rat, for example, mounting by the male produces tactile stimulation of the flanks, posterior rump, perineum, and tail base, which in turn activates the external clitoris and clitoral inputs to the pudendal nerve (Kow & Pfaff, 1973). As the male intromits, pressure receptors in the vagina, cervix, and uterus are also stimulated, which in turn activates the hypogastric and pelvic nerves. Similar nerves are activated in the male during intromissions and ejaculation. Genital stimulation reaches the spinal cord mainly at the lumbosacral level (Giuliano & Rampin, 2004; Lee & Erskine, 1996) although a secondary pathway has been suggested via the vagal nerves bypassing the spinal cord in humans (Komisaruk et al., 2004). In both cases, ascending inputs may activate hypothalamic neurons directly via the spinohypothalamic pathway (Cliffer, Burstein, & Giesler, 1991) or at the hindbrain and cerebellum via the spinothalamic, dorsal column–medial lemniscus and dorsal spinocerebellar pathways, respectively (Cliffer et al., 1991; Okahara & Nisimaru, 1991; Sengul, Fu, Yu, & Paxinos, 2015), specifically in the nucleus of the tractus solitarius (NTS), midbrain periaqueductal gray matter (PAG), and cerebellar vermis (Menetrey & Basbaum, 1987; Young, Murphy Young, & Hammock, 2005) (Berkley et al., 1988; Berkley, Robbins, & Sato, 1993; Manzo et al., 2008; Mouton, VanderHorst, & Holstege, 1997; Paredes-Ramos, Pfaus, Miquel, Manzo, & Coria-Avila, 2011). The NTS and PAG project to hypothalamic areas such as the medial preoptic area (mPOA), ventral hypothalamus, and paraventricular nucleus (PVN) (Marson & Murphy, 2006). The PVN contains parvocellular neurons that produce OT and AVP, which are important pro-bonding neurotransmitters (Lim & Young, 2004). The PVN also projects to the medial amygdala (MeA) and releases OT to facilitate social recognition (Ferguson, Aldag, Insel, & Young, 2001) but may also induce learning of stimuli that will facilitate sexual motivation by stimulating the activity of the mPOA, bed nucleus of the stria terminalis (BNST), and the hippocampus (Been & Petrulis, 2011; Yang, Oberlander, & Erskine, 2007). Small regions within the MeA, mPOA, BNST, and cerebellar vermis respond selectively to ejaculation in males (Coolen, 2005; Manzo et al., 2008) or paced copulation and clitoral stimulation in females (Erskine & Hanrahan, 1997; Parada et al., 2010; Paredes-Ramos et al., 2011). With regard to the cerebellum, it can project information via thalamus to the motor cortex (Sommer, 2003), frontal cortex (Middleton & Strick, 2001), midbrain, pons, and back to the spinal cord (Bastian, 2006; Glickstein & Doron, 2008; Ito, 2006; Ramnani, 2006). Activation of the cerebellum yields changes of activity in the septal region, hippocampus, and amygdala (Heath, Dempesy, Fontana, & Myers, 1978). Altogether, these areas are important for detecting the differences in VCS during paced or non-paced copulation, timed CLS, and during ejaculation, playing a role in the processing of sexual reward and learning (Fig. 1).

**Fig. 2:**
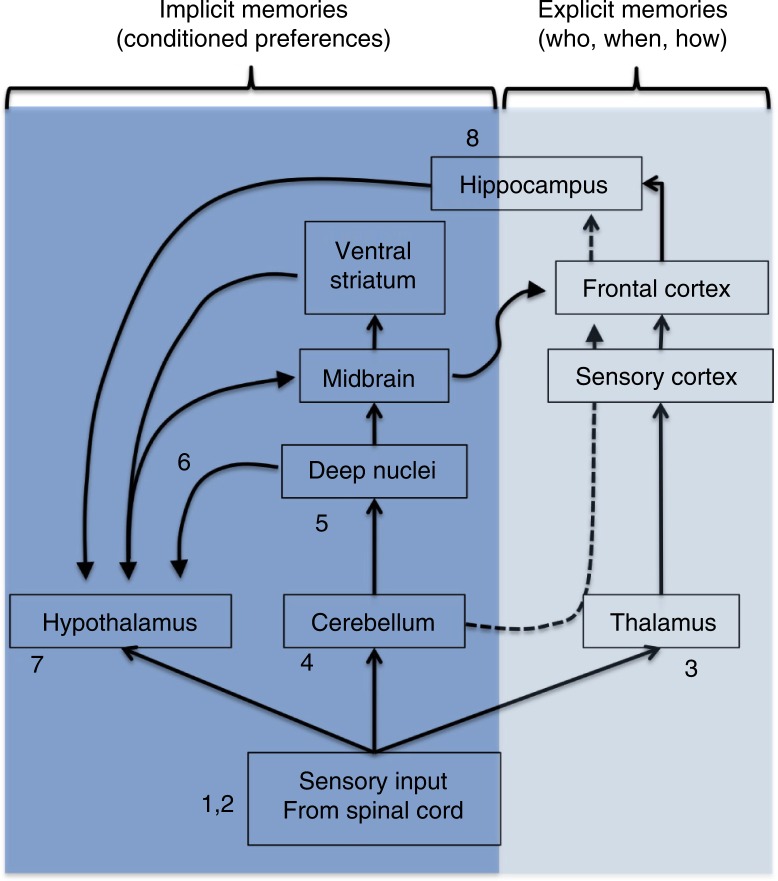
Central neurocircuitry involved in the development and shaping of orgasm-induced partner preferences. For full explanation, see Section ‘A model’. (1, 2) Sensory input projects to thalamus; (3) from thalamus to the sensory cortex where orgasm is experienced as pleasurable, and associative cortices where explicit memories of Who, When, and How the orgasm was experienced are processed. (4) Spino-cerebellar pathway projects information to the cerebellum where implicit memories may be processed in coordination with cortices. (5) Deep nuclei, the main cerebellar output to midbrain and hypothalamus. (6) The cerebellum-hypothalamic pathway. (7) Hypothalamus mediates sexual reward and motivation, and along with the medial amygdala may process social recognition and motivation. (8) Hippocampus may facilitate the crystallization between the experience of orgasm and cues on a partner. It can influence motivation via the mPOA to the midbrain ventral tegmental area (VTA), affecting the level of dopaminergic activity in ventral striatum (nucleus accumbens).

Other brain areas that mediate motivation and learning become more active during a sexual encounter. For example, in the nucleus accumbens (NAc) of the ventral striatum, the levels of dopamine (DA) increase in males during mounts with intromission and fall rapidly after ejaculation (Blackburn, Pfaus, & Phillips, 1992; Fiorino, Coury, & Phillips, 1997), whereas in female rats, DA levels increase during paced copulation and fall when the male is taken away (Becker, Rudick, & Jenkins, 2001; Mermelstein & Becker, 1995). Although DA release may not contribute directly to the ejaculation- or pacing-induced reward state, it has been argued that it may convey qualitative or interpretive information about the rewarding value of stimuli (Becker et al., 2001), particularly during the pre-orgasmic state. Such dynamics in DA release become important in some species (i.e. monogamous) during the development or shaping of partner preferences, since DA antagonists (i.e. haloperidol) – either into the NAc or systemically injected prior to copulation – disrupt the partner preference formation that would occur after copulation, whereas low doses of DA agonists (i.e. apomorphine), or specific D2-type (but not D1-type) receptor agonists, facilitate pair bonding even without copulation (Aragona, Liu, Curtis, Stephan, & Wang, 2003; Gingrich, Liu, Cascio, Wang, & Insel, 2000; Wang et al., 1999). In the NAc, there is a simultaneous interaction of DA and OT during the formation of partner preferences, since blockade of either receptor with infusions of antagonists prevents the development of pairbonds induced by copulation (Liu & Wang, 2003; Young, Lim, Gingrich, & Insel, 2001). Interestingly, OT also mediates the rewarding experience induced by opioids (Moaddab, Hyland, & Brown, 2015), such that under the effects of OT, animals are more likely to display opioid-induced past learning. Indeed, in many studies, it has been argued that it is not the intensity of an orgasm that modulates the probability of development of a pair bond but rather the natural expression of OT, AVP, and D2 receptors in those brain circuits (i.e. as observed in monogamous vs. polygamous voles). Nevertheless, studies carried out in non-monogamous species, like rats, indicate that repeated copulation with a partner can facilitate the development of a partner preference. This suggests that exposure to the same partner during orgasm in males and females can possibly sensitize brain areas that mediate bonding, even in species that would normally not bond, like rats (Fig. 2).

## Neural correlates in humans

Functional magnetic resonance imaging (fMRI) and positron-emission tomography (PET) have been used to measure brain activity during orgasm in humans (Georgiadis et al., 2006; Georgiadis, Reinders, Van der Graaf, Paans, & Kortekaas, 2007; Komisaruk & Whipple, 2005; Komisaruk et al., 2004). Like in rats, ejaculation in men activates the cerebellum (deep dentate nucleus, anterior vermis), pons, and ventrolateral thalamus, whereas it deactivates the prefrontal cortex (Georgiadis et al., 2007). In women, orgasm also activates the cerebellum (left deep nuclei), the ventral midbrain, right caudate nucleus, hippocampus, and medulla oblongata (Komisaruk & Whipple, 2005). As in men, orgasm in women decreases activity in the neocortex, particularly in the left lateral orbitofrontal cortex, inferior temporal gyrus, and anterior temporal pole (Georgiadis et al., 2006). The areas activated during orgasm may also be activated in future encounters as implicit memories and conditioned responses during observation of cues that have been paired with orgasm in the past, facilitating retrieval of episodic memories and partner preferences (Fig. 2). For example, studies with fMRI have shown that bonded individuals that observe the lover's picture respond with increased activity in areas associated with memory such as the dentate gyrus in the hippocampus (Bartels & Zeki, 2004), involved in the processing of episodic memory. The ventral region of the anterior cingulate cortex (ACC) is also activated with the lover's picture (Bartels & Zeki, 2004), probably related to autonomic responses like heart rate and blood pressure control but also cognitive functions like reward anticipation and discrimination of stimuli (Parkinson, Cardinal, & Everitt, 2000). The ventral tegmental area (VTA) (at the midbrain) and the medial insula also increase their activity (Bartels & Zeki, 2004). The VTA is the site of origin of mesolimbic DA neurons that project to limbic structures, including the NAc, MeA, and ACC, and is probably responsible for increased motivation and attention, whereas the medial insula may explain visceral sensations induced by the picture of the beloved.

The increased brain activity during sexual reward in humans and animals (Komisaruk & Whipple, 2005; Kurtz, 1975; Yang et al., 2007) suggests an ongoing process of reward, attention, and memory consolidation (both explicit and implicit), and its subsequent recovery during exposure to partner-related cues (Bartels & Zeki, 2004). As mentioned above, the hippocampus is activated during orgasm and during picture exposure. This structure expresses AVP receptors that mediate pair bonding (Sofroniew, Weindl, Schrell, & Wetzstein, 1981). In addition, during copulation of female rats, the hippocampus responds with high-frequency electrical theta activity that increases during solicitations and slows down during mounts and intromissions, and becomes of high-amplitude during the end of a copulatory session, when sexual satiety is likely to occur (Kurtz, 1975). In males, high-amplitude hippocampal theta activity is mainly observed during the PEI when the male appears to be drowsy (McIntosh, Barfield, & Thomas, 1984). Interestingly theta hippocampal activity can also be induced by intra-brainstem injections of opiates (i.e. morphine) (Leszkowicz, Kusmierczak, Matulewicz, & Trojniar, 2007), suggesting that the post-orgasmic release of endogenous opioids mediates hippocampal theta activity. Post-learning sleep facilitates memory consolidation, especially during high-amplitude and synchronized slow oscillations (Bodizs, Bekesy, Szucs, Barsi, & Halasz, 2002). Accordingly, during the post-orgasmic period, an individual's brain is in ‘learning mode’ (Figs. 1 and 2).

## Pharmacological correlations

Experiencing an orgasm does not guarantee the development of a partner preference. This may occur for many different reasons, among which are natural modifications of the neurochemical mechanisms of bonding (as described in monogamous voles) or as a consequence of pharmacological manipulations or emotional states that alter those mechanisms. For instance, monogamous voles that have already bonded with a partner will not form a new preference with a second partner regardless of the opportunity to copulate or cohabit. This occurs mainly as a result of D1-type receptor upregulation in the NAc after the first pair bond (Aragona et al., 2006; Young & Wang, 2004). Within the NAc, D1-type activation prevents the formation of pair bonds, whereas D2-type activation facilitates their formation (Aragona et al., 2006). Thus, a single vole requires enhancement of D2-type receptor activity to form a new partner preference, which is followed by enhanced D1-type activity to prevent more bonding with additional partners. Interestingly, drugs of abuse like amphetamines enhance D1-type receptor activity, resulting in the incapacity of voles and humans to develop pair bonds (Liu et al., 2010; Syvertsen et al., 2015). Other drugs of abuse, like methylenedioxy methamphetamine (MDMA or ‘Ecstasy’), may enhance levels of serotonin to a point where it prevents males from learning via association with sexual reward (Straiko, Gudelsky, & Coolen, 2007). This probably occurs as a result of the exaggerated serotonergic activity in the NAc and other brain areas such as the mPOA, MeA, VTA, and PAG following MDMA injections (Thompson, Hunt, & McGregor, 2009). Opioid receptor agonists also alter the natural mechanisms of reward, which helps to explain how opiate abuse may impair orgasm and orgasm-induced partner preferences. Opiates cause euphoria in humans and are self-administered in virtually all animals. Depending on the type of opioid receptor activated, opiates can facilitate or inhibit DA release in the NAc via the VTA (Di Chiara & Imperato, 1988). The disinhibition of DA neurons by µ or d opioids arriving from the arcuate nucleus to the ventral tegmental area (VTA) inhibits gamma-aminobutyric acid (GABA) neurons (Mucha & Herz, 1985) that results in the activation of VTA DA neurons which project to, and release DA into, the NAc (Herz, 1998). Furthermore, a subpopulation of DA neurons from the VTA to the NAc also possesses κ receptors in NAc terminals, which inhibit DA release (Heijna et al., 1990). Thus, DA release may facilitate or prevent pair bonding depending on what DA receptor type is mainly present in the individual (D2 vs. D1). Accordingly, some individuals may experience powerful orgasms and not be able to develop pair bonds or partner preferences, whereas others may develop them during their early experiences with sexual reward.

## A model

Based on the references cited above, we depict a model of the basic neural pathways that participate during the experience of orgasm and the resulting development of partner preference (Figs. 1 and 2). (1) During sex, the sensory input activates the pudendal, pelvic, and hypogastric nerves to reach the spinal cord at the L6-S1 level. Ejaculation in males is mediated by a spinal control center, referred to as a spinal pattern generator (not shown in figures) that coordinates sympathetic, parasympathetic, and motor (somatic) outflows, integrating the latter with the inputs from supraspinal sites in brainstem, hypothalamus, and preoptic area. Clitoral stimulation activates spinal inputs via the pudendal nerve that activates a similar pattern of supraspinal sites. These ultimately induce a pattern of vaginal tenting along with cervical and uterine contractions that accompany the experience of orgasm in females. (2) The sensory input projects via de dorsal column–medial lemniscus pathway and spinothalamic tract (not shown in figures) to the thalamus. (3) From the thalamus, information projects to the sensory cortex where orgasm is experienced as pleasurable, and explicit memories of Who, When, and How the orgasm was experienced are processed. (4) At the same time, the spino-cerebellar pathway projects information to the cerebellum, which is highly active during orgasm, suggesting that sexual reward may be also processed to some extent in the cerebellum. (5) The cerebellar Purkinje neurons project to cerebellar deep nuclei, which are the main cerebellar outputs. (6) Accordingly, cerebellar activity can reach the hypothalamus via the cerebellum-hypothalamic pathway. (7) Hypothalamic nuclei such as the paraventricular, medial preoptic (mPOA), and BNST may be the main mediators between sexual reward and motivation, and along with the MeA may process social recognition and motivation. Some hypothalamic nuclei can be directly activated via the spino-hypothalamic pathway (not shown in Fig. 1). (8) After orgasm, the hippocampus expresses high-amplitude theta frequency that is believed to facilitate episodic memory consolidation, probably facilitating the crystallization between the experience of orgasm and cues on a partner. The hippocampus projects back to the hypothalamus via the fornix, where it can influence motivation via the mPOA to the midbrain VTA, affecting the level of dopaminergic activity in nucleus accumbens. (9) In addition to these pathways, the vagus nerves can function as a second pathway for orgasmic sensory input as observed in spinal cord injured persons. (10) The vagus can project to the NTS and to the periaqueductal gray matter (PAG), which also project directly to the hypothalamic areas.

## Conclusions

When an individual receives the appropriate appetitive, precopulatory, and copulatory stimulation, orgasm (or an orgasm-like response in animals) is more likely to occur. In males and females, orgasm/reward functions as a potent Pavlovian UCS. Some areas in the nervous system function as UCS-detection centers, which become activated during orgasm. Partner-related cues (e.g. odors and strains) may function as CSs and are processed in CS-detection centers. In order to develop an orgasm-induced partner preference or a pair bond, UCS- and CS-detection centers must interact to facilitate memory consolidation and produce recognition and motivation during future social encounters. It has become apparent that the activation of endogenous opioid systems is the basic neurochemistry for orgasm/sexual reward. DA and other monoamines set arousal and attention, and drive appetitive/consummatory behaviors, whereas OT and AVP set contact, consummatory behaviors, and bonding. In humans, this process may account for the crystallization of individual ‘types’ that are sought out as partners, based on a conglomeration of distal, proximal, and interactive cues (Pfaus et al., 2012). It may also account for the fixation on inanimate objects or other fetish behaviors that were present and acted upon during an individual's first experience with sexual arousal and/or pleasure (De Block & Adriaens, 2013; Gross, 2014; Krafft-Ebing, 1894; Pfaus, Erickson, & Talianakis, 2013).
